# The continuous performance test aids the diagnosis of post-stroke cognitive impairment in patients with right hemisphere damage

**DOI:** 10.3389/fneur.2023.1173004

**Published:** 2023-06-28

**Authors:** XiuLi Li, FuBiao Huang, TieJun Guo, MengChen Feng, Shan Li

**Affiliations:** ^1^Faculty of Rehabilitation, Capital Medical University, Beijing, China; ^2^Department of Occupational Therapy, China Rehabilitation Research Center, Beijing, China

**Keywords:** functional near-infrared spectroscopy (fNIRS), continuous performance test, poststroke cognitive impairment, prefrontal cortex, neural compensatory responses

## Abstract

**Purpose:**

The purpose of the study was to investigate the time course difference of relative changes in oxygenated hemoglobin (Oxy-Hb) concentration in the prefrontal cortex (PFC) between controls and patients with post-stroke cognitive impairment (PSCI) who had right hemisphere damage (RHD) using the continuous performance test (CPT) and functional near-infrared spectroscopy (fNIRS) technology. The study aimed to evaluate the feasibility of CPT in the diagnosis and evaluation of PSCI with RHD.

**Methods:**

A total of 16 patients with RHD (RHD group) and 32 normal subjects (control group) were recruited. The Montreal Cognitive Assessment Scale was used to assess post-stroke cognitive impairment. The CPT and fNIRS were employed to investigate task-related changes in Oxy-Hb levels.

**Results:**

The RHD group showed significantly lower accuracy and hit rates than the control group; however, the average reaction time was significantly longer in the former. Although the two groups showed no statistically significant difference in terms of left and right PFC integral values, the mean values were greater in the RHD group. The centroid value of the right PFC was significantly higher in the RHD group than in the control group. The time course of Oxy-Hb concentrations in the PFC differed between the two groups. In the RHD group, neural compensation was observed in both prefrontal lobes; however, the rate of compensation was slower on the affected side.

**Conclusion:**

The CPT may be helpful in the clinical diagnosis of PSCI with RHD. It may therefore be used to evaluate the effectiveness of cognitive interventions.

## 1. Introduction

Most stroke patients suffer from varying degrees of cognitive damnification (1). Post-stroke cognitive impairment (PSCI), a subtype of vascular cognitive impairment, is a common and serious consequence of stroke. It seriously affects functional recovery, increases the medical burden on families and society, hinders return to family and society, and reduces the quality of life (2–4). Studies suggest that 56 to 79% of patients experience impairment in ≥1 cognitive domains following a stroke (1). Early identification, prevention, and treatment of PSCI are essential for rehabilitation of stroke patients and for improving their prognosis. The prediction and diagnosis of PSCI is usually performed using clinical and neuropsychological assessments (5), such as Mini-Mental State Examination (MMSE) and Montreal Cognitive Assessment (MoCA). However, due to fairly limited sensitivity and inability to detect specific subdomains, these scales can lead to missed diagnosis, especially in individuals with early mild cognitive impairment (MCI).

Functional near-infrared spectroscopy (fNIRS) is a non-invasive portable optical functional brain imaging modality, which offers good cost effectiveness and high ecological validity. It can provide more friendly and relaxed trial conditions, that make patient-focused clinical cognitive studies more simple, convenient, and feasible. It has also become an important tool for assessing task-related cerebral oxygenation and autoregulation in real time during cognitive tasks. The level of brain function can be evaluated indirectly by determining the time course of physiological and pathological changes in the Oxy-Hb response of the cerebral cortex. Studies have shown that brain perfusion-based or oxygenation-based biomarkers may be important factors in assisting the diagnosis and prediction of psychiatric disorders, moderate cognitive impairment, and dementia (6–9). Task-based fNIRS can be used to explore cognitive function in various diseases; it can also be used to evaluate differences between patients with disease and normal control groups (10–12). Stroke patients may demonstrate an abnormal hemodynamic response when the brain performs a cognitive task. In this context, a previous study discovered no significant difference between patients with stroke and healthy individuals in terms of the PFC hemodynamic response (13). However, no studies have investigated the hemodynamic response during fNIRS in patients with PSCI who have right hemisphere damage (RHD). In addition, the continuous performance test (CPT) is not used as a cognitive activation task during fNIRS in patients with PSCI and RHD. It also remains unclear whether the time course and sensitivity of bilateral PFC hemodynamic responses differ between RHD and control groups.

Notably, the frontal lobe is crucial in the formation, maintenance, and recovery of cognitive function. As a multi-functional optical neuroimaging technology, fNIRS can be used to investigate the hemodynamic response of the PFC in neurological conditions (14–16). The CPT, as an attention task, has been used to detect task-related PFC function (17, 18); in this context, attention function is the basis of cognitive function. Compared with high-level cognitive functions such as memory, attentional function is affected in a higher proportion of stroke patients (19, 20). More than 80% of patients demonstrate values below the normal cutoff in at least one domain of attention (21). Compared with other cognitive task paradigms, CPT may be more suitable for identifying patients with PSCI.

In this study, CPT and task-state fNIRS were used to compare brain activity between patients with PSCI who had RHD and healthy controls. The neural compensatory mechanism of PSCI with RHD was also studied.

## 2. Materials and methods

### 2.1. Participants

Overall, 16 patients with PSCI and RHD who had been admitted to the Occupational Therapy Department of the Beijing Boai Hospital between January 2021 to May 2022 were included in this study. Thirty-two age-matched healthy volunteers with MoCA score of >26 were selected for inclusion in the control group (Table 1). The inclusion criteria of the RHD group were: (1) conforming to the diagnostic criteria of stroke proposed at the Fourth National Conference on Cerebrovascular Diseases (these patients with stroke having right hemisphere damage at first onset were also diagnosed using computed tomography or magnetic resonance imaging of the brain) (22), (2) meeting the diagnostic criteria of PSCI (5), and (3) having a MoCA score of<26 (23), (4) 14–150 days elapsed since stroke, (5) aged 18–71 years, (6) having no complicating neurodegenerative diseases, (7) having no history of mental illness, and (8) had provided informed consent. The exclusion criteria were: (1) having cognitive impairment before stroke, (2) taking medications that may affect the results of the assessment, (3) sensory aphasia and severe cognitive dysfunction (with MoCA scores of<14) that could prevent completion of assessment (24), (4) presence of cranial defects, and (5) a history of alcoholism. The current study was approved by the China Rehabilitation Research Center Ethics Committee (approval number: 2021-015-1).

**Table 1:** General demographic characteristics of the two groups.

Variables	Control group(*n* = 32)	RHD group (*n* = 16)	*z/t/χ* ^2^	*p*
Age (years)	48.53 ± 9.09	52.50 ± 7.63	1.500	0.140
Sex^a^ (M/F)	19/13	13/3	2.297	0.130
Years of education	13.84 ± 3.04	14.13 ± 3.16	0.298	0.767
MoCA	30.00 (30.00,30.00)	19.00 (18.00,20.75)	−6.356	≤0.001
Handedness^b^ (R/L)	32/0	16/0	–	–

a Sex: M, Male; F, Female.

b Handedness: R, Right handedness; L, Left handedness.

### 2.2. Assessment of cognitive function

The MoCA scale is one of the most widely used tools for assessing cognitive function. It contains several domains including executive function, naming, attention, language, abstraction, delayed recalls, and orientation. The Beijing version, which has good reliability and validity, was used in this study. The Cronbach’s α of the Beijing version of the MoCA scale was 0.752; the correlation coefficients between executive function, attention, abstraction, delayed recalls, orientation, and total scores were 0.664 ~ 0.763; the correlation coefficient between MMSE and MoCA scores was 0.975 (25–27).

### 2.3. Experimental procedure

**Figure 1:**
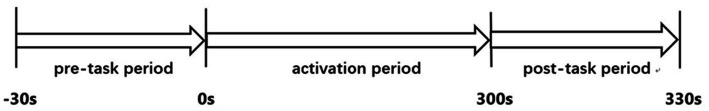
The continuous performance test protocol. A 30 s pre-task baseline period, a 300 s activation period, and a 30 s post-task baseline period were combined.

The cognitive paradigm used in this study for cortex activation was the CPT. It took the subjects 360 s to complete the test. This included a pre-task baseline period of 30 s, an activation period of 300 s, and a post-task baseline period of 30 s (Figure 1).

### 2.4. Activation task

The X version of CPT (Japanese Society for Advanced Brain Function) was used in this study and a computer was used to present the stimulant. The numbers 1–9 were displayed randomly to the participants on a computer screen and number 7 was the target number. The numbers were displayed in white font in the center of a black screen. The stimuli were presented at a fixed rate of once every 1,000 ms. There were 24 and 96 target and non-target stimuli, respectively. After being comfortably seated, the subjects were instructed to press the space bar with their healthy index finger as soon as they saw the number 7. Although the version of CPT used in this study was the Japanese version, the details of the test were explained to the subjects in Chinese. As the presented stimuli were all numbers, subject’ understanding was not affected.

### 2.5. fNIRS measurements

**Figure 2:**
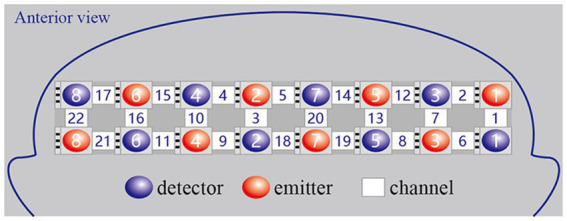
The setting of optodes.

**Figure 3:**
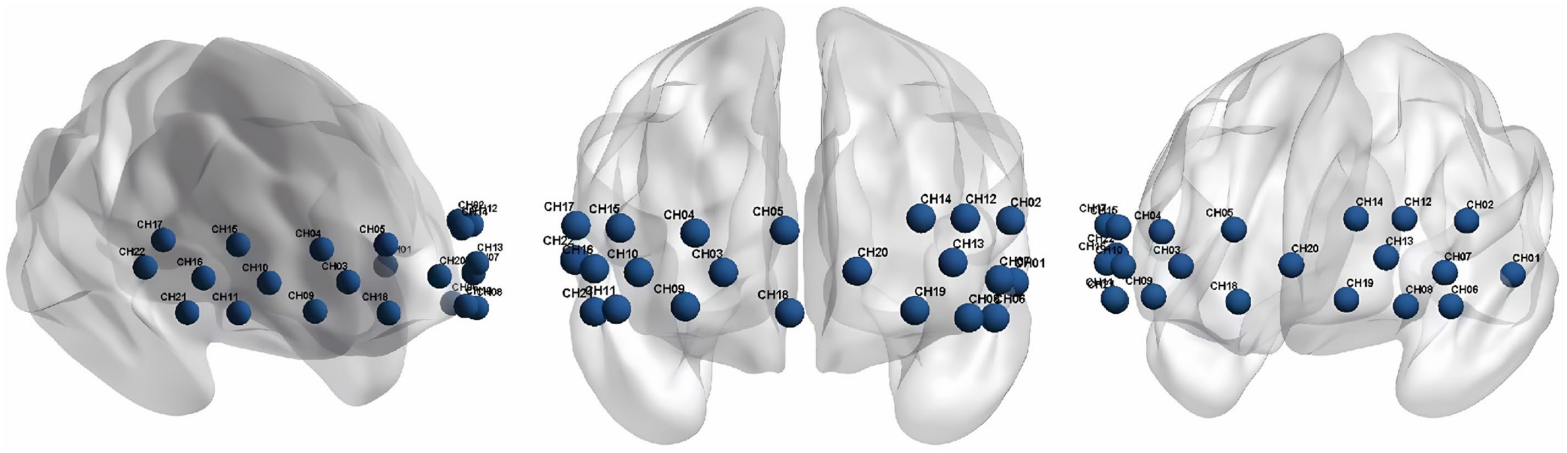
The 3-dimensional location of the 22 channels.

We used a continuous wave and portable fNIRS system (LIGHTNIRS; Shimadzu Co.) at 3 wavelengths (780 nm, 805 nm, and 830 nm) to measure the changes in Oxy-Hb concentrations. The 8 emitters and 8 detectors were arranged in a 2×8 array, placed on a light elastic cap and positioned on the prefrontal lobe (Figure 2). According to the international 10–20 electrode configuration method, the bottom probes were located along the Fp1–Fp2 line. There was a distance of 3cm between each emitter and detector pair. In this study, the midpoints of the corresponding emitter-detector pairs were defined as the fNIRS channels. There were 22 channels in total (Figure 3). The Fpz was positioned at the midpoint of the bottom probe connection line, while ensuring that the probe set was placed over the two regions of interest, namely, the left and right PFC. The 2 middle channels, 5 and 18, were removed during data processing. The left PFC was covered by channels 1, 2, 6, 7, 8, 12, 13, 14, 19, and 20 and the right PFC was covered by channels 3, 4, 9, 10, 11, 15, 16, 17, 21, and 22. The sampling rate was 13.33 Hz.

### 2.6. Data pre-processing

As various approaches have been reported for the analysis of fNIRS data, no standardized method has been established. The Homer2 open source software in MATLAB was used for analyzing the data pertaining to the fNIRS images. The processing steps were as follows: (1) the raw data (light intensity) were converted into the optical density, (2) artifacts were identified and bad channels were marked manually, (3) automatic artifact recognition and interpolation correction were used to correct artifacts of the data; channels with obvious motion artifacts were considered as bad channels and excluded from further analysis, (4) the optical density was converted into the blood oxygen concentration, and (5) a band-pass filter (at 0.003 ~ 0.1 Hz) was used to remove physiological noise (i.e., the heart rate, respiratory rate, and low frequency physiological fluctuations of less than 0.01 Hz); this was performed to eliminate high frequency machine noise and slow wave drifts.

Alterations in Oxy-Hb levels were observed to be more sensitive and reliable than those of deoxy-hemoglobin for evaluating regional cerebral blood flow (28). Only the Oxy-Hb data were analyzed in this study. The last ten seconds of the pre-task period was used as the baseline period for baseline correction. Alterations in Oxy-Hb levels relative to baseline were calculated during CPT. The average changes in Oxy-Hb were calculated for each individual per channel and the integral and centroid values were calculated from these variables. The data from the left and right PFC channels were then, respectively, averaged. The integral value was defined as the sum of Oxy-Hb concentrations from the beginning to end. The centroid value was represented by time, which was denoted by the vertical line on the centroid of the fNIRS signal change region over all task periods.

### 2.7. Statistical analysis

A patient’s data were considered valid if six of the ten channels in each hemisphere were available for statistical analysis. The independent samples t-test was used to test significant differences between the RHD-PSCI and control groups. The level of statistical significance was set at *p* < 0.05.

## 3. Results

The fNIRS data of all subjects from both groups were included for the final statistical analysis.

### 3.1. Demographics

The demographic data are shown in Table 1. No significant differences were observed between the two groups in terms of age, sex, years of education, and handedness (*p* > 0.05). The RHD group showed significantly lower MoCA scores than the control group (*p* < 0.001).

### 3.2. Behavioral results during CPT

The RHD group showed significantly lower accuracy and hit rates than the control group (*p* < 0.05). The average reaction time was significantly longer in the RHD group than in the other group (*p* < 0.05; Table 2).

**Table 2:** Comparison of behavioral results between control and RHD groups.

Variables	Control group(*n* = 32)	RHD group (*n* = 16)	*z*/*t*	*p*
Accuracy rate	100.00 (100.00–100.00)	100.00 (95.83–100.00)	−1.999	0.046
Hit rate	100.00 (100.00–100.00)	96.35 (91.10–100.00)	−2.546	0.011
Average reaction time	510.61 ± 47.95	573.43 ± 110.95	−2.166	0.044

### 3.3. Centroid and integral values

There was a statistically significant difference between the RHD and control groups in terms of right PFC centroid values. However, no statistically significant difference was observed between the two groups in terms of left and right PFC integral values. Nevertheless, the mean left and right PFC integral values were higher in the RHD group than in the other group (Table 3).

**Table 3:** Results of centroid and integral values.

Hemisphere	Control group (*n* = 32)		RHD group (*n* = 16)		Integral values		Centroid values	
	Integral values	Centroid values	Integral values	Centroid values	*t*	*p*	*t*	*p*
Left PFC	−1.32E-01 ± 1.97E+00	166.13 ± 22.57	1.83E-01 ± 2.59E+00	167.16 ± 24.09	−0.469	0.641	−0.145	0.885
Right PFC	−1.92E-01 ± 1.91E+00	159.15 ± 21.02	2.60E-01 ± 2.43E+00	175.26 ± 26.66	−0.705	0.485	−2.282	0.027

### 3.4. Time course of oxy-Hb concentration changes

**Figure 4:**
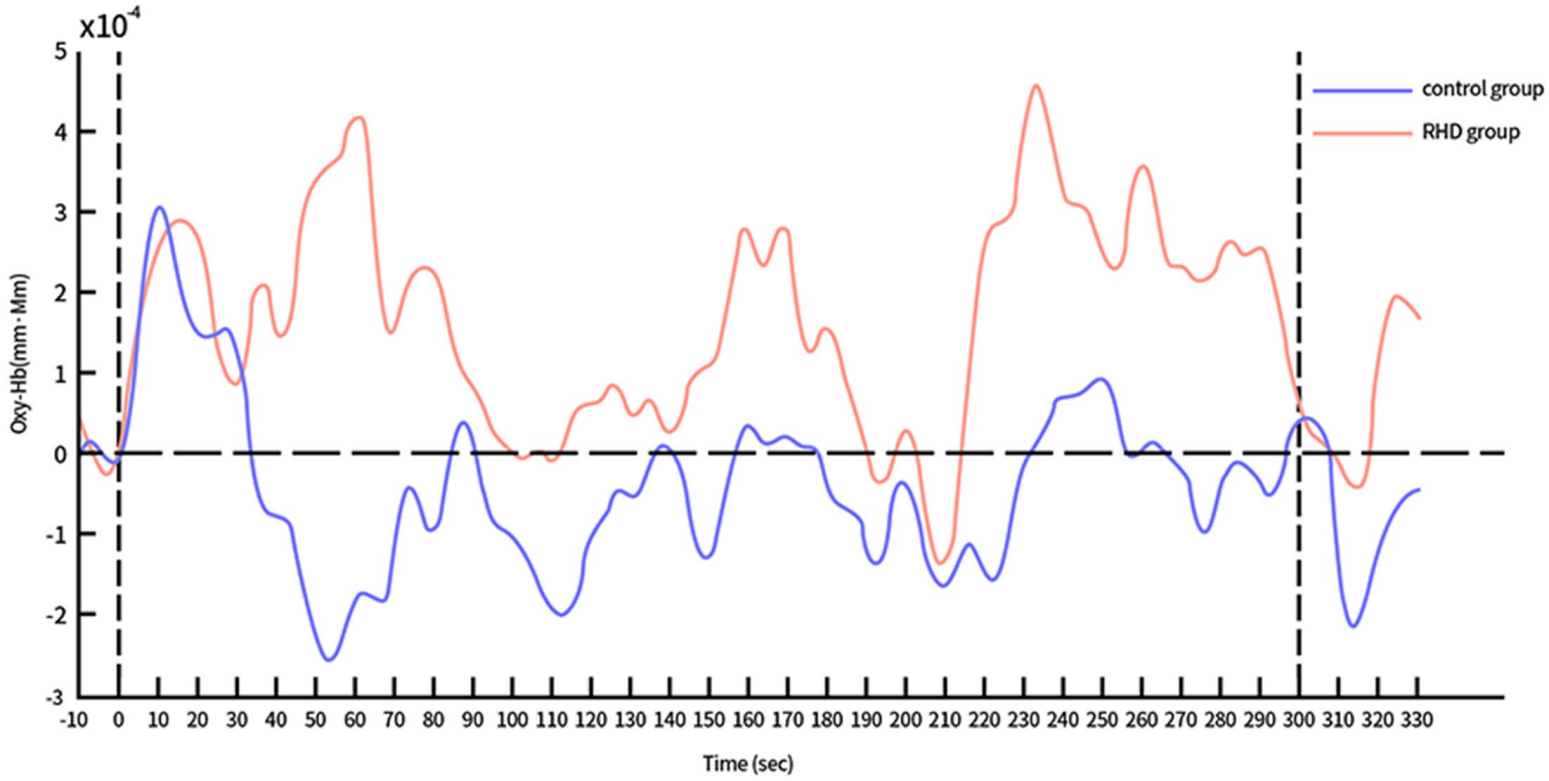
Time course of Oxy-Hb concentration changes in the left PFC during CPT (in both control and RHD groups).

**Figure 5:**
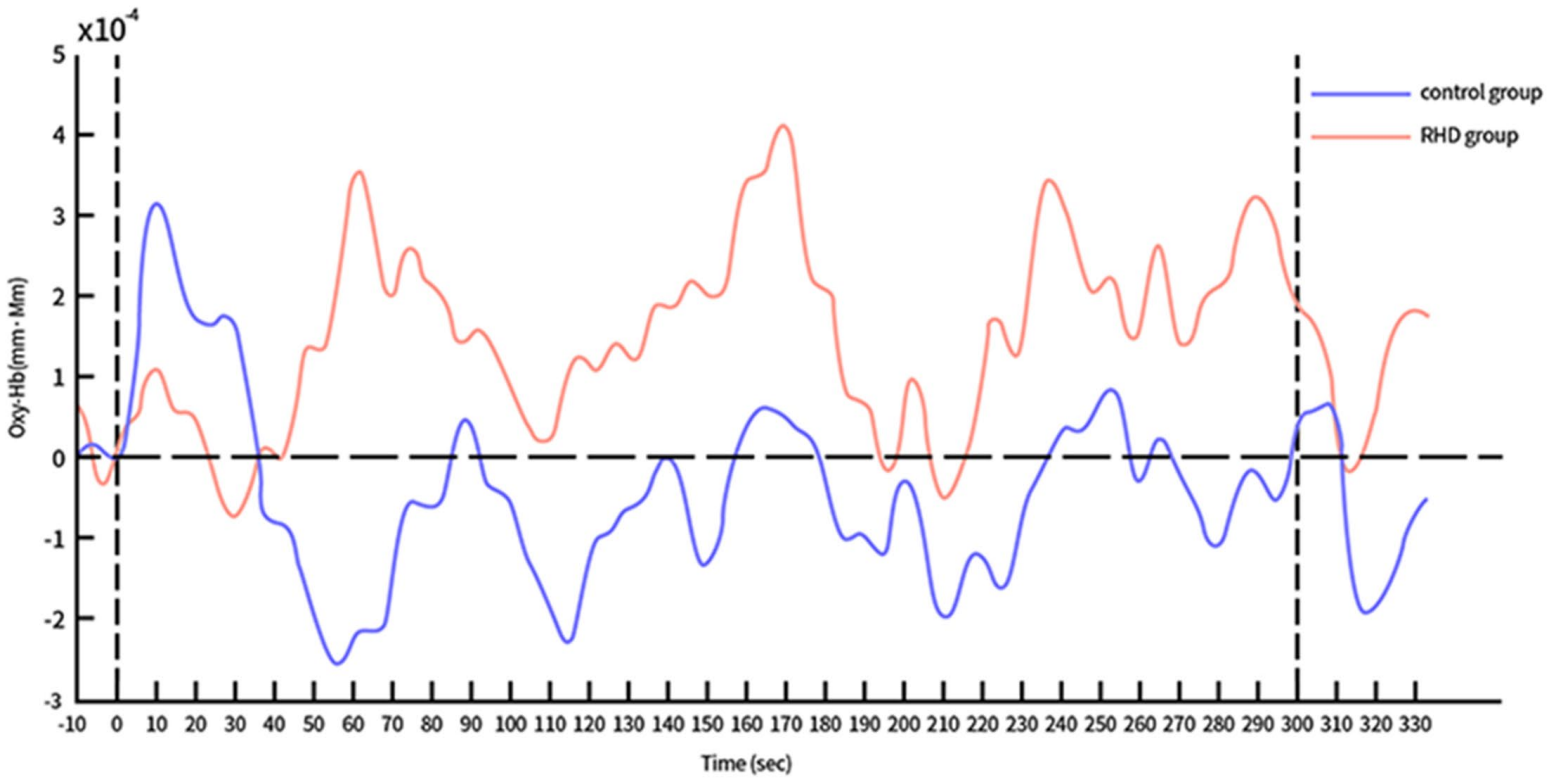
Time course of Oxy-Hb concentration changes in the right PFC during CPT (in both control and RHD groups).

The time course of Oxy-Hb concentration changes differed significantly between the control and RHD groups (Figures 4, 5). Prefrontal Oxy-Hb concentrations rose rapidly in both groups after the task began and reached the first peak at approximately 10 s after task initiation; it then declined gradually. However, the first activation peak in the right PFC was smaller in the RHD group than in the other group. There was no obvious Oxy-Hb activation in the control group approximately 35 s later. In the RHD group, bilateral PFCs reactivated multiple times after 35 s, with greater activation than in the control group; this also exceeded the first peak of activation.

## 4. Discussion

This study evaluated the fNIRS of bilateral PFC Oxy-Hb concentrations during the CPT in both, patients with PSCI who had RHD and healthy controls. The time series of Oxy-Hb values demonstrated differences between patients and controls; the group with PSCI and RHD showed increased bilateral prefrontal activation compared with the control group. The activation rate of the affected PFC was slower in patients with PSCI.

In the study, the time course of Oxy-Hb concentration changes differed between the control and RHD groups (Figures 4, 5). Oxy-Hb concentrations rose rapidly during the task in both groups, reaching the first peak at approximately 10 s after the task began. The Oxy-Hb level in the control group then began to decline, with no further significant Oxy-Hb responses in bilateral prefrontal lobes until the end of the task. This may be attributed to the fact that normal people find the CPT easy. They mastered and adapted to it within the first few tens of seconds of testing and began to relax and had less cognitive needs. In this context, reduced cognitive needs may result in a lower hemodynamic response than before the test. Conversely, there were several significant activation responses in the RHD group and the activation level also exceeded the maximum value of the first activation; this increase continued until the task was completed. Studies show that the degree of activation increases with an increase in the difficulty and cognitive demands of the task (29). Our study showed similar results; the multiple activations after the first activation peak may be attributed to the fact that the extension of time increases the cognitive load of CPT. In their study, Hironori et al. found the time series of Oxy-Hb concentration changes to differ significantly between older and younger groups after increasing the task load using dual tasks (30): levels of Oxy-Hb increased gradually in older individuals and the increase continued until the dual task was completed. However, Oxy-Hb levels gradually decreased in the younger group (after the first peak of activation) until the completion of the task. Both RHD and control groups in this study demonstrated a similar time course. Notably, the findings of bilateral increased activation in patients with PSCI and RHD were consistent with the compensatory mechanism indicated by the revised Scaffolding Theory of Aging and Cognition regulatory model (31). According to the revised Scaffolding Theory of Aging and Cognition regulatory model, healthy older adults experience greater structural degeneration and functional deterioration of the brain than younger adults (32). In order to ameliorate or counteract the adverse effects of neural and functional decline, the aging brain is provided the necessary additional computational support via compensatory scaffolding; this allows maintenance of cognitive function during neural and functional decline (32). Literature on neuroimaging suggests that over activation or additional recruitment of prefrontal brain regions are evident indications of compensatory scaffolding (33, 34). Patients with PSCI and RHD also showed greater activation and bilateral recruitment of the prefrontal lobe relative to healthy controls, with compensatory mechanisms similar to those seen in healthy aging populations.

Our results also showed slower and less efficient right (affected side) PFC compensation in the RHD group compared to the other group. This suggests that the nerve processing speed and quality had decreased in the affected cerebral hemisphere. It was necessary to recruit further healthy PFC neurons to compensate for the reduced processing efficiency. It may therefore be appropriate to distinguish between injury-measured and non-injury-measured cerebral hemispheres when exploring changes in cognitive training-induced brain activation in patients with PSCI. Notably, the right cerebral hemisphere may be a key stimulation site for future interventions using non-invasive brain stimulation (such as transcranial magnetic and direct current stimulation) in patients with PSCI and RHD.

The cognitive task paradigm used had certain characteristics. First, the task period of this study was relatively long. A 300 s task period was used to examine the detailed time course of Oxy-Hb responses in the prefrontal lobe. The time course of changes in Oxy-Hb concentrations demonstrated a persistent difference between the RHD and other group throughout the task. Second, the CPT used was easily comprehensible and had low difficulty. Unlike other cognitive activation tasks that have a higher demand for memory function, this task could be performed easily by patients with PSCI; they could quickly adapt, master, and cooperate to complete the task. Third, the CPT used was more suitable for solitary cognitive activation of the frontal lobe, because it required minimal motor responses (space bar pressing) and no eye movements. This study showed that the use of a long (300 s) simple cognitive activation task can induce compensatory activation in patients with PSCI who have RHD; it can also provide a fuller and more detailed characterization of the time course of the frontal Oxy-Hb response.

Studies using CPT and fNIRS to compare outcomes of cognitive activation in patients with PSCI and RHD are currently lacking. An increased cognitive load through prolonged cognitive testing could explain the results in healthy controls and these patients. The reduced cognitive processing efficiency of the affected PFC in these patients also resulted in an increase in PFC centroid values. Based on our preliminary results, the differences in prefrontal responses between the control and RHD groups during the CPT could be used in clinical settings to assist in the identification of patients with PSCI and RHD.

### 4.1. Limitations

The small sample size was the major limitation of this study. In addition, as the detection range of fNIRS is limited by the number of probes, it is not possible to measure all cortical activation changes in the brain. Although a chinrest was used during the test to limit head movements (to reduce the effect of head movements on fNIRS signals) as far as practicable, factors such as skin blood flow and skull thickness could have potentially influenced the fNIRS signals. Future studies are therefore needed to address the relationship between fNIRS signals and these confounding factors. The findings from our preliminary study suggests that meaningful fNIRS findings may be obtained in patients with PSCI and RHD. Further studies with larger sample sizes are warranted for validating these findings. Future research on multimodal fusion will also be needed to identify better approaches for clinical practice, including the integration of fNIRS and electroencephalography or functional magnetic resonance imaging.

## 5. Conclusion

This study showed cognition-related activation changes in bilateral PFCs in both control and RHD groups during the CPT. Compensatory activation was observed in bilateral prefrontal lobes of patients with PSCI and RHD. However, the rate of compensatory activation was slower on the affected side. The time course of Oxy-Hb concentration changes also differed between the two groups. The CPT, which is closely related to brain cognitive function, is expected to be an auxiliary diagnostic tool for identifying PSCI in patients with RHD. It may also be used as a tool to evaluate the effect of cognitive interventions.

## Data availability statement

The raw data supporting the conclusions of this article will be made available by the authors, without undue reservation.

## Ethics statement

The studies involving human participants were reviewed and approved by the China Rehabilitation Research Center Ethics Committee (approval number: 2021-015-1). The patients/participants provided their written informed consent to participate in this study.

## Author contributions

XL and FH designed the study. XL analyzed the data and wrote the manuscript. XL, SL, TG, and MF performed data collection and literature review. All authors contributed to the article and approved the submitted version.

## Funding

This study was supported by the Fundamental Research Funds for Central Public Welfare Research Institutes (2019CZ-11) and the Project of China Rehabilitation Research Center (number: 2021zx-Q4).

## Conflict of interest

The authors declare that the research was conducted in the absence of any commercial or financial relationships that could be construed as a potential conflict of interest.

## Publisher’s note

All claims expressed in this article are solely those of the authors and do not necessarily represent those of their affiliated organizations, or those of the publisher, the editors and the reviewers. Any product that may be evaluated in this article, or claim that may be made by its manufacturer, is not guaranteed or endorsed by the publisher.
